# Sensitive screening of single nucleotide polymorphisms in cell free DNA for diagnosis of gestational tumours

**DOI:** 10.1038/s41525-022-00297-x

**Published:** 2022-04-08

**Authors:** Geoffrey J. Maher, Rosemary A. Fisher, Baljeet Kaur, Xianne Aguiar, Preetha Aravind, Natashia Cedeno, James Clark, Debbie Damon, Ehsan Ghorani, Adam Januszewski, Foteini Kalofonou, Ravindhi Murphy, Rajat Roy, Naveed Sarwar, Mark R. Openshaw, Michael J. Seckl

**Affiliations:** 1 Trophoblastic Tumour Screening & Treatment Centre, Imperial College London, Charing Cross Campus, Fulham Palace Road, London, W6 8RF UK; 2 Department of Surgery and Cancer, ICTEM Building, Hammersmith Hospitals Campus of Imperial College London, Du Cane Road, London, W12 0NN UK

**Keywords:** Cancer genetics, Medical genetics

## Abstract

Tumours expressing human chorionic gonadotropin (hCG), the majority of which are difficult to biopsy due to their vascularity, have disparate prognoses depending on their origin. As optimal management relies on accurate diagnosis, we aimed to develop a sensitive cell free DNA (cfDNA) assay to non-invasively distinguish between cases of gestational and non-gestational origin. Deep error-corrected Illumina sequencing of 195 common single nucleotide polymorphisms (SNPs) in cfDNA and matched genomic DNA from 36 patients with hCG-secreting tumours (serum hCG 5 to 3,042,881 IU/L) and 7 controls with normal hCG levels (≤4 IU/L) was performed. cfDNA from confirmed gestational tumours with hCG levels ranging from 1497 to 700,855 IU/L had multiple (*n* ≥ 12) ‘non-host’ alleles (i.e. alleles of paternal origin). In such cases the non-host fraction of cfDNA ranged from 0.3 to 40.4% and correlated with serum hCG levels. At lower hCG levels the ability to detect non-host cfDNA was variable, with the detection limit dependent on the type of causative pregnancy. Patients with non-gestational tumours were identifiable by the absence of non-host cfDNA, with copy number alterations detectable in the majority of cases. Following validation in a larger cohort, our sensitive assay will enable clinicians to better inform patients, for whom biopsy is inappropriate, of their prognosis and provide optimum management.

## Introduction

**Fig. 1:**
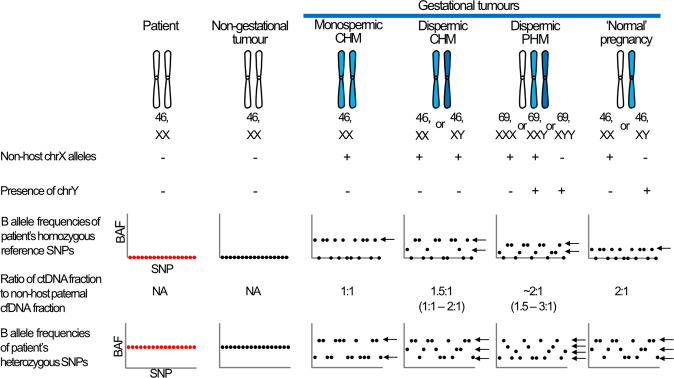
Expected cfDNA profiles from tumours of different origins. Gestational tumours have chromosomes from the father of the pregnancy (blue). Monospermic complete hydatidiform moles are androgenetic, diploid and homozygous, due to absence of maternal DNA and duplication of a haploid sperm’s genome. Dispermic complete hydatidiform moles are diploid androgenetic, having two chromosome complements of paternal origin. Dispermic partial hydatidiform moles are triploid diandric, have two paternal chromosome complements and one maternal complement. Normal pregnancies are diploid, having one paternal and one maternal complement. The B allele frequencies (BAFs) (y axis) of each SNP (x axis) in cfDNA (black) from non-gestational tumours matches that of the patient’s gDNA (red), although the heterozygous SNPs may deviate from 50:50 due to copy number alterations (not shown). Due to differences in their genetic composition, cfDNA from gestational tumours display non-host alleles at different fractions and have different profiles for a patient’s homozygous and heterozygous SNPs, the latter of which may only be discernible at relatively high fractions of ctDNA. Thus, the non-host (paternal) cfDNA fraction may underestimate the ctDNA fraction. The presence of chrY and non-host chrX alleles is indicative of an XY or XXY dispermic molar pregnancy.

Gestational trophoblastic tumours (GTTs) comprise a group of malignancies that arise from products of conception. The tumours, which include invasive mole, choriocarcinoma and the rarer placental site and epithelioid trophoblastic tumours (PSTT and ETT), may arise from pre-malignant hydatidiform moles (abnormal pregnancies caused by an excess of paternal DNA^1^) or from normal, ectopic, miscarried, or aborted pregnancies (Fig. 1)^2^. The majority of GTTs arising from molar pregnancies are diagnosed during routine monitoring of serum human chorionic gonadotrophin (hCG) levels in the months following evacuation of the molar pregnancy. However, GTTs arising from molar and other types of pregnancy may occur years after the causative pregnancy, by which point the disease has, in many cases, metastasised to the lungs, brain or other tissues^3^. Although hCG is a highly sensitive marker of trophoblastic disease that correlates with tumour volume^3^, this marker is also expressed by some non-gestational malignancies^4^.

Determining the origin of hCG producing tumours in women is crucial for optimal patient management and counselling as, although GTTs are highly curable, the prognosis for patients with non-gestational tumours is invariably poorer^5^. Furthermore, patients with hCG-secreting non-gestational tumours can avoid inappropriate intensive anti-cancer therapy if they can be reliably identified. Short-tandem repeat (STR) genotyping of tumour and normal tissue is typically used to distinguish between the two entities; gestational tumours uniquely have alleles from the paternal component of the causative pregnancy, but the alleles of non-gestational tumours match those of the patient^6^. Due to the vascular nature of trophoblastic tumours, biopsies may result in life-threatening haemorrhage and are not routinely performed when a gestational tumour is suspected.

Previous studies have demonstrated that circulating tumour DNA (ctDNA) is detectable in cell-free DNA (cfDNA) circulating in GTT patients’ plasma, but the approaches have limitations which preclude their use for diagnostics^7–9^. STR genotyping can be applied to any case but lacks sensitivity, failing to detect ctDNA when serum hCG is lower than ~15,000 international units per litre (IU/L) and with variable detection up to 60,000 IU/L^7^. The highly sensitive duplex sequencing approach used by Lavoie et al. requires DNA from the father of the pregnancy and the design of custom patient-specific probes targeting rare single nucleotide polymorphisms (SNPs) identified in a prior sequencing experiment^9^. In addition to the limitation that the father of the pregnancy may not be known or be available for testing, these requirements substantially increase expense and turnaround time, precluding its routine use. To overcome the limitations of both methods we devised a common SNP-based assay that is sensitive, applicable to the majority of patients, and only requires their blood sample making it suitable for routine diagnostics.

## Results

### Validation using ctDNA of known origin

As gestational tumours uniquely contain DNA from the paternal component of the causative pregnancy (Fig. 1), we screened 195 common autosomal SNPs (minor allele frequencies ranging from 0.26 to 0.71) to determine if ‘non-host’ alleles were present in plasma cfDNA of female cancer patients with elevated serum hCG. Illumina sequencing of gDNA was used to identify homozygous SNPs in the patient and simultaneous ultradeep sequencing (median read depth ~8500×) of the cfDNA samples (Supplementary Table 1)) was used to detect the presence or absence of non-host alleles at these loci. The use of unique molecular identifiers facilitated error correction and quantification of the number of molecules assessed (by generating consensus reads; median 635 per cfDNA sample), which is important to ensure that sufficient theoretical sensitivity is achieved for samples such as cfDNA which typically have low input amounts (~10–20 ng) and may have low proportions of the DNA population of interest (<10%)^10–12^.

**Fig. 2:**
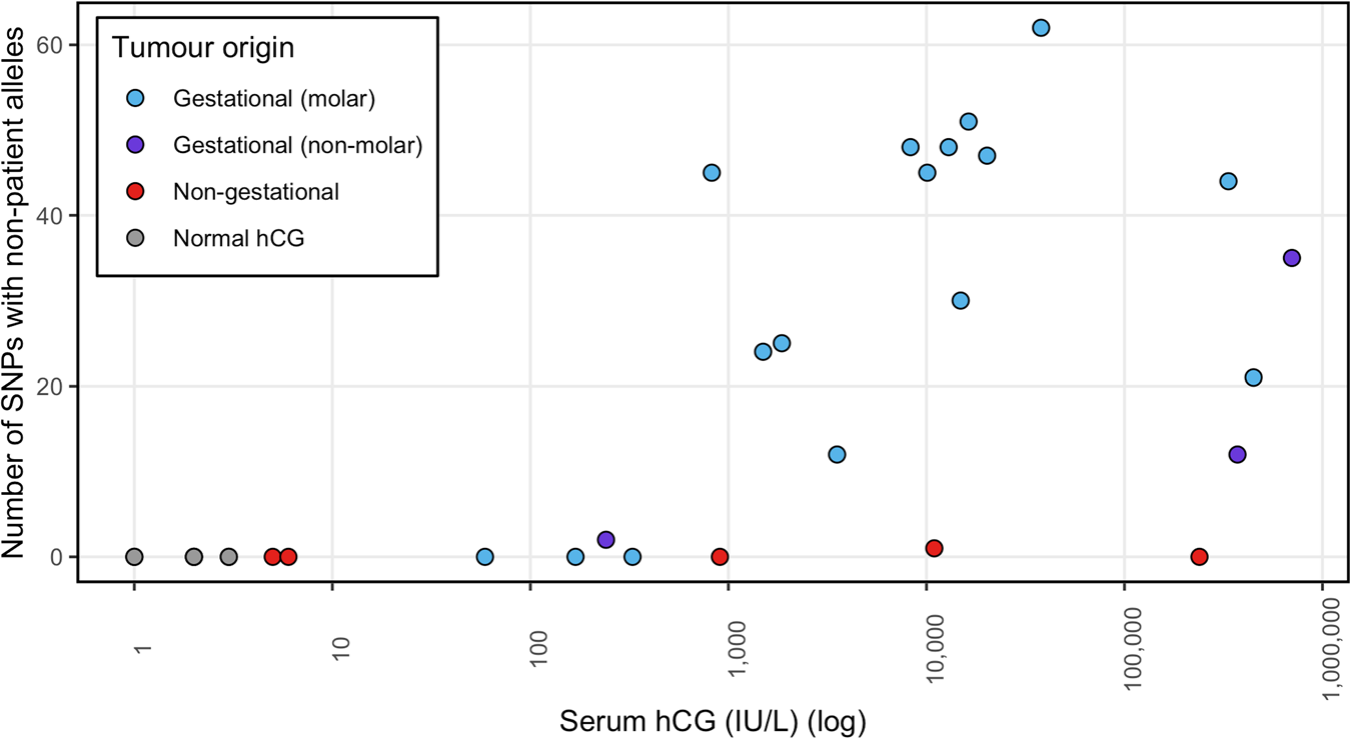
Number of SNPs with non-host alleles in cfDNA from controls and tumour cases of known origin. Gestational tumour patients had ≥12 SNPs with non-host alleles when hCG levels ≥824 IU/L, but only 0-2 had detectable non-host alleles when hCG levels were ≤328 IU/L. Samples from patients with non-gestational tumours or normal hCG (≤4 IU/L) levels had 0 or 1 SNP with non-host alleles.

To assess the sensitivity and specificity of the assay, we screened gDNA and cfDNA samples from gestational tumour cases with serum hCG levels ranging from 59 to 700,855 IU/L (*n* = 19) and compared results to those from females with non-gestational tumours (hCG range 5–239,171 IU/L) (*n* = 5) and females with normal hCG levels (≤4 IU/L) (*n* = 7). Whilst the genotype of cfDNA from a non-gestational tumour is expected to match that of the patient, cfDNA from a gestational tumour is expected to carry non-host (i.e. paternal) alleles at approximately half of the SNPs where the patient is homozygous (Fig. 1). As expected, no non-host alleles were detected in the cases with normal hCG levels (Fig. 2). Four of the five non-gestational cases had no non-host alleles and one case (GTD018) had a single SNP with a low-frequency variant (2/411 consensus reads) that did not match the patient. Thus, in total, only 1 of the 1211 homozygous SNPs across the 12 patients (median of 101 per patient) had an allele in cfDNA that did not match the respective gDNA sample. In gestational cases where the hCG was ≥824 IU/L, multiple SNPs (mean 36; range 12–62) had non-host alleles, but when the hCG ≤ 328 IU/L the number of SNPs with non-host alleles was in the range of non-gestational cases (0–2 SNPs) (Fig. 2). IU/L

A genomic DNA sample from the father of the causative pregnancy was available for four gestational cases. All of the non-host alleles identified in the patients’ cfDNA were present in the respective paternal sample, confirming their gestational origin (Supplementary Fig. 1). Although allele frequencies for each independent SNP varied within a patient, analysis of separate cfDNA aliquots of the same sample extracted four years apart showed that allele frequencies for each SNP were consistent (Supplementary Fig. 1 A, B), demonstrating that targeting multiple SNPs gives a reliable method of detection and estimation of the proportion of non-host cfDNA. The non-patient alleles detected in samples from two distinct timepoints for CFD007 were also consistent (Supplementary Fig. 1C).

Probes targeting seven chrX loci (including four common SNPs) and six chrY loci were used to determine the sex of the tumour. Reads aligning to chrY were detected in the single case known to have originated from a normal male pregnancy (CFD031)^7^ and in three of the 17 cases derived from molar pregnancies (CFD003, CFD010, CFD015), in cases with non-host fractions as low as 0.5%. As dispermic XY or XXY molar pregnancies harbour paternally inherited X chromosomes but normal XY pregnancies do not, it is possible to distinguish between the two entities using chrX SNPs (Fig. 1). Even though the assay was underpowered to identify such occurrences as only four common chrX SNPs were analysed, non-host chrX alleles were detected in two of the three dispermic XY cases (CFD003 and CFD010) (Table 1). Analysis of B-allele frequencies confirmed the monospermic molar origin of CFD007 and suggested CFD002 may have a similar origin (Supplementary Fig. 2).

**Table 1:** Detection of non-host cfDNA in plasma of patients with hCG-secreting tumours of known origin.

Patient	Serum hCG (IU/L)	Days since first treatment	Tumour Origin	Mean consensus read depth	Number of SNPs with non-host alleles	Non-host allele fraction (mean ± sd)	Sex chr
NP05	5	13	Non-gestational	756	0	NA	X
CFD017	6	6	Non-gestational	1263	0	NA	X
GTD025	906	93	Non-gestational	979	0	NA	X
GTD018	10953	2	Non-gestational	610	1	NA	X
CFD008	239171	10	Non-gestational	728	0	NA	X
GTD008	59	4	Gestational (molar)	464	0	NA	X
CFD028	169	0	Gestational (molar)	453	0	NA	X
GTD013	241	172	Gestational (non-molar)	410	2	0.5 ± 0.1%^a^	X
GTD017	328	2	Gestational (molar)	538	0	NA	X
GTD022	824	2	Gestational (molar)	495	45	1.3 ± 0.7%	X
CFD005	1497	3	Gestational (molar)	679	24	0.5 ± 0.3%	X
GTD005	1861	0	Gestational (molar)	553	25	0.7 ± 0.4%	X
GTD006	3537	0	Gestational (molar)	614	12	0.4 ± 0.2%	X
CFD007b	5501	6	Gestational (molar)	547	20	2.1 ± 0.7%	X
CFD012	8308	2	Gestational (molar)	597	48	5.7 ± 2.9%	X
CFD001	10097	1	Gestational (molar)	782	45	1.4 ± 0.8%	X
CFD002	12946	6	Gestational (molar)	782	48	8.4 ± 2.1%	X
CFD009	14884	6	Gestational (molar)	874	30	3.3 ± 1.0%	X
CFD010	16326	3	Gestational (molar)	697	51	7.3 ± 2.5%	X + Y
CFD015	20237	6	Gestational (molar)	1053	47	4.5 ± 2.1%	X + Y
CFD003	37923	6	Gestational (molar)	909	62	1.6 ± 1.2%	X + Y
NP06	335039	13	Gestational (molar)	1021	44	30.4 ± 3.8%	X
CFD023	372117	0	Gestational (non-molar)	1001	12	13.2 ± 3.7%	X
CFD007	448650	21	Gestational (molar)	339	21	41.4 ± 10.0%	X
CFD031	700855	1	Gestational (non-molar)	656	35	28.1 ± 8.5%	X + Y

Cases are listed by origin and increasing hCG levels. Further details are provided in Supplementary Table 1.

*NA* not applicable.

^a^Likely to be an overestimate due to failure to detect the majority of non-host alleles.

### Copy number alterations in non-gestational cases

**Fig. 3:**
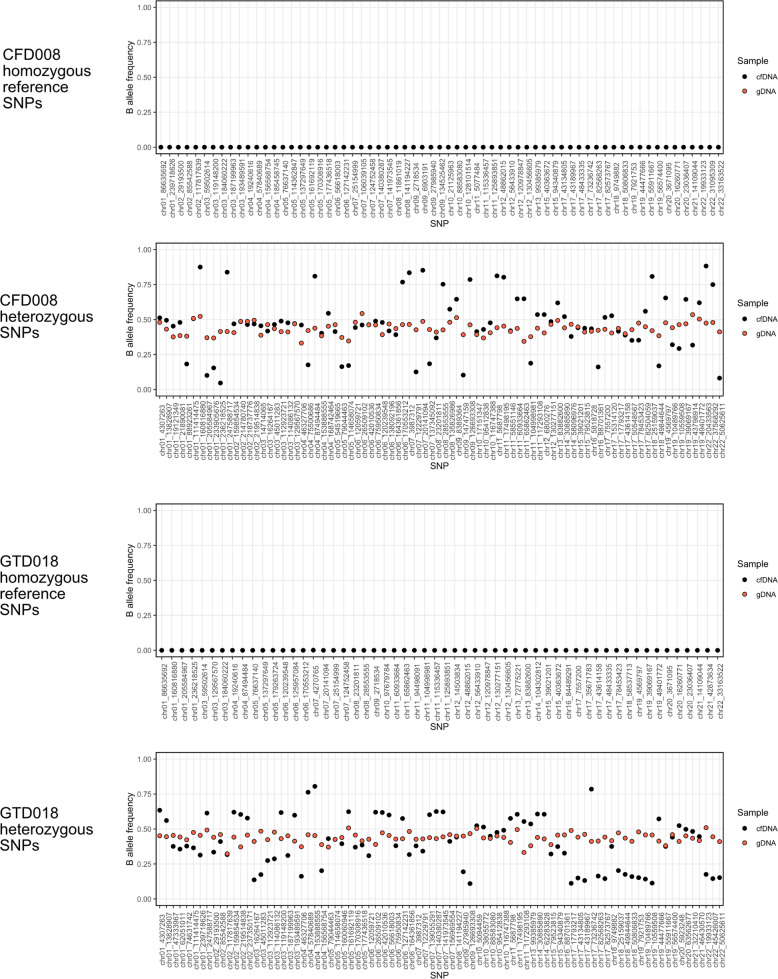
Copy number alterations in non-gestational tumour cfDNA. Upper panels for each patient show the absence of non-host alleles at SNPs for which the patient is homozygous. Lower panels for each patient show the B allele frequency for heterozygous SNPs, in order of chromosome and position, revealing deviations from the patients’ gDNA sample.

The absence of non-host alleles in a cfDNA sample from a patient with elevated hCG could be due to failure to detect very low levels of gestational ctDNA (e.g. in cases with low hCG levels) or due to the tumour being non-gestational. The presence of chromosomal copy number alterations (CNAs) has previously been reported in some non-gestational tumours^6,13^ and in cfDNA from CFD008 by STR genotyping^7^. We proposed that CNAs may provide an alternative means to confirm the presence of non-gestational ctDNA in samples which lack evidence of non-host alleles and that these could be detected by identifying deviation of the B allele frequency of SNPs from the heterozygous state. The B allele profiles of heterozygous SNPs in the cfDNA from CFD008 (hCG 239,171 IU/L) and GTD018 (hCG 10,953 IU/L) had evidence of CNAs at multiple chromosomes (Fig. 3), but these were not found in the three cases with low hCG levels less than 1000 IU/L.

### Cases without a prior diagnosis

After demonstrating the validity of the assay, we subsequently screened a further twelve patients with a hCG-secreting tumour (range 242–3,042,881 IU/L) of unknown origin i.e. patients who at the time of sampling did not have a tissue biopsy, only two of which had a known history of molar pregnancy. A single blood sample was taken from each patient within the first 16 days of undergoing chemotherapy and three patients (NP01, NP02, NP03) had longitudinal sampling (Table 2 and Supplementary Table 1). A DNA sample was available from the partner of only two patients (NP08 and NP09).

**Table 2:** Detection of non-host cfDNA in plasma of patients with hCG-secreting tumours of unknown origin.

Patient	serum hCG IU/L	Days since first treat-ment	Mean consensus read depth	Number of SNPs with non-host alleles	Non-host allele fraction (mean ± sd)	Sex chr	Tumour origin based on cfDNA	Tissue diagnosis (post-cfDNA)^a^
CFD014	5254	4	658	19	0.6 ± 0.3%	X	Gestational	Not available
GTD002	3042881	0	965	42	40.5 ± 14.3%	X	Gestational	Not available
GTD009	3840	2	488	1	NA	X	Non-gestational	Non-gestational
GTD028	131783	1	886	0	NA	X	Non-gestational	Non-gestational
NP01	463901	6	225	50	18.9 ± 4.0%	X + Y	Gestational	Not available
NP01b	242	32	790	3	0.3 ± 0.0%^b^	X	Undetermined	Not available
NP02	5262	16	562	41	1.0 ± 0.5%	X + Y	Gestational	Not available
NP02b	437	31	1046	0	NA	X	Undetermined	Not available
NP03	60899	0	898	44	2.7 ± 1.2%	X + Y	Gestational	Not available
NP03b	1174	17	784	3	0.2 ± 0.0%^b^	X + Y	Undetermined	Not available
NP04	906	13	747	33	1.0 ± 0.6%	X	Gestational	Gestational
NP07	28400	0	1650	42	9.6 ± 3.1%	X	Gestational	Gestational
NP08	128328	0	825	34	9.8 ± 2.7%	X	Gestational	Not available
NP09	127874	0	444	0	NA	X	Non-gestational	Non-gestational
NP10	23913	3	859	15	0.3 + 0.1%	X	Gestational	Not available

Samples are ordered by serum hCG levels. Further sample details are provided in Supplementary Table 1.

*NA* not applicable.

^a^Tumour samples were subsequently available for GTD009, GTD028, NP04, NP07 and NP09 and the cfDNA diagnosis was confirmed by STR genotyping of the matched tumour.

^b^Likely to be an overestimate due to failure to detect the majority of non-host alleles.

All patients except GTD009, GTD028 and NP09 (discussed below) had multiple SNPs (median 39; range 15–50) with non-host alleles in their primary plasma cfDNA samples (Table 2 and Supplementary Fig. 3). The gestational nature was supported by the presence of chrY reads in three cases (NP01, NP02, NP03) and a B-allele frequency profiles in concordance with those of monospermic and dispermic molar pregnancies in NP08 and GTD002, respectively (Supplementary Fig. 2). Patients NP04 and NP07 subsequently had tumour tissue available for STR genotyping, which confirmed the gestational origin (Supplementary Fig. 4).

To investigate the sensitivity of the assay, we analysed additional samples from NP01, NP02 and NP03 when the patients’ tumour biomarker (hCG) levels had dropped by 1–3 orders of magnitude to 242, 437, and 1174 IU/L, respectively. None of the non-host alleles previously identified at 41 SNPs in NP02 were detected and only 3/50 and 3/44 non-host alleles were detected for NP01 and NP03, respectively (Table 2). As only ~25% of each sample library was pooled for probe capture of the SNP regions, we subsequently pooled, captured, and sequenced the remainder of the libraries for these three samples with the aim of increasing the number of unique molecules and thus the sensitivity. Despite lowering the limit of detection (0.23%, 0.12% and 0.18% for NP01b, NP02b and NP03, respectively), the number of SNPs with non-host alleles only marginally increased to four and five for NP01b and NP03b, respectively, while non-host alleles remained undetectable for NP02b (Supplementary Table 1). This suggests that majority of the non-host alleles in these samples with low hCG levels were present at levels below 0.2% of the total cfDNA.

**Fig. 4:**
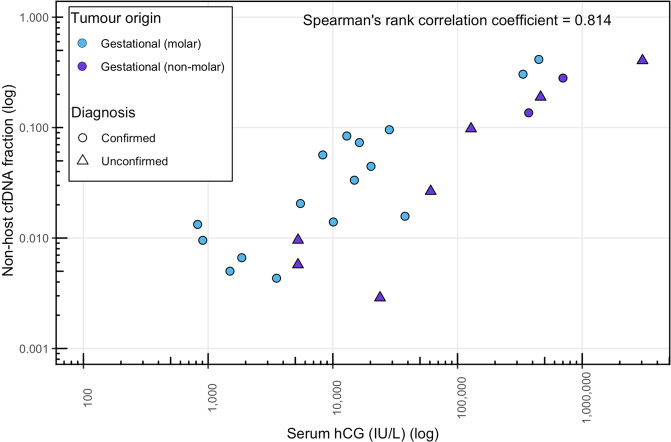
Non-host allele fractions in cfDNA compared to serum hCG levels in gestational tumour cases. Non-host cfDNA fractions tend to be relatively higher in tumours originating from molar pregnancies than other pregnancies as they contain two paternally inherited genomes. Only samples with reliable estimates of cfDNA fraction (i.e. ≥12 SNPs with non-host alleles) were included. Confirmed = cases with a known gestational origin. Unconfirmed = cases with non-host cfDNA that did not have tissue available to confirm the diagnosis.

Overall, the serum hCG level of gestational samples correlated with the fraction of non-host cfDNA (Spearman’s rho = 0.814, *P* = 2.4 × 10^−6^) (Fig. 4). Based on their hCG levels, the ctDNA fraction in GTD028 and NP09 is expected to be >10%, but non-host alleles were not detected at any of their homozygous SNPs, consistent with non-gestational tumours. GTD009 had a relatively low hCG level (3840 IU/L) and had a single SNP with non-host alleles at 0.5% allele frequency (3/639 consensus reads). All three patients subsequently had tumour tissue removed; STR genotyping confirmed their non-gestational origin with presence of copy number alterations (data not shown), indicating that the single SNP with a non-host allele in GTD009 was due to a low-frequency error or a somatic mutation. These and other copy number alterations were identifiable as allelic imbalances in all three cfDNA samples and the only available tumour sample (Supplementary Fig. 5). Although direct comparison between gestational and non-gestational ctDNA fractions could not be made, the amount of cfDNA per ml of plasma was generally relatively higher in non-gestational cases (Supplementary Fig. 6).

## Discussion

In addition to the clinical benefit due to the risks associated with taking tumour biopsies and the difference in prognosis and management compared to non-gestational tumours, GTT are ideally suited for ctDNA diagnosis due to their unique genetic make-up. The presence of paternal DNA from the causative pregnancy provides a wide and highly-specific target for detection, circumventing the typical requirement of knowledge of tumour-specific recurrent mutations or patient-specific mutations for other solid tumours^14,15^ and avoiding confounding events such as clonal haematopoiesis^16^.

Our assay confidently detected non-host cfDNA with fractions as low as 0.28% (Fig. 4), the lowest reported to date in trophoblastic tumour samples. In gestational cases derived from molar pregnancies, ≥12 SNPs with non-host alleles were detected in cases with hCG levels as low as 824 IU/L. However, in the cases without a history of molar pregnancy (thus likely originating from normal pregnancies), when the hCG was 1174 IU/L, or lower, non-host cfDNA was only detectable at <10% of the expected SNPs. The difference in molar and normal pregnancy cases can be attributed in part to the different relative contributions of paternal DNA (two paternal genomes in molar pregnancies and one in normal pregnancies) (Fig. 1) as demonstrated by molar pregnancies typically having relatively higher non-host cfDNA fractions (Fig. 4). Although the duplex sequencing assay appeared to have greater sensitivity than our assay based on hCG levels (732 IU/L) the fraction of non-host cfDNA was ~0.8%, which would have been detectable with our assay. Thus it is likely that other factors such as tumour type, that can vary in their hCG secretion^17^, tumour site, and time since treatment could also affect the ratio between hCG and non-host cfDNA fraction.

Across seven normal hCG and eight non-gestational samples, only two out of a total of 1502 homozygous SNPs had a false positive non-host allele present at low levels, indicating that the error rate of this consensus-read based assay is low. We conservatively set the detection threshold of 12 SNPs with non-host alleles, which had consistent results when hCG levels were ≥1497 IU/L, but further sampling of gestational and non-gestational cases will likely reduce this threshold and determine whether other informative features (e.g. amount of cfDNA per ml (Supplementary Fig. 6)) can be used to distinguish between gestational and non-gestational cases when hCG levels are low. Although copy number alterations detected using the B allele profile of the heterozygous SNPs can confirm the presence of ctDNA in non-gestational cases (Fig. 3), such events were not detectable in the cases with hCG levels <1000 IU/L.

Despite the technical limitations at low hCG levels, using the first sample taken after admission we could determine the origin of all 12 of the test cases (Table 2) without the need for a partner’s DNA sample. In all seven patients who were clinically treated as gestational cases and did not have surgery or a biopsy during their treatment, non-host alleles were detected. Their assumed gestational origin was further supported by the detection of a Y chromosome in 3 cases (Table 2), and B-allele frequency profiles indicative of molar origin in two cases (Supplementary Fig. 2). In the absence of tissue to confirm the diagnosis, sequencing cfDNA from a distinct plasma sample or using digital PCR or alternative techniques^18^ to screen a subset of the identified informative SNPs could be utilised to independently confirm the findings. Identifying the causative pregnancy may help to further stratify patients as the type and interval since the causative pregnancy is a factor in the FIGO/WHO risk scoring of gestational trophoblastic tumours^19^, with the latter being the most important prognostic factor for PSTT and ETT^20,21^.

Owing to the rarity of fresh tumour material, there have been few genome-wide studies of GTT^22,23^ particularly at base-pair resolution^24^ and no recurrent or tumour subtype-specific mutations have been identified. Thus, although we demonstrate that ctDNA can be effectively used for diagnosis of gestational tumours, specifying the tumour subtype remains elusive. In conclusion we have developed a sensitive, reproducible, and affordable (~$220 per case) non-invasive diagnostic technique for identifying GTT which can be used for any patient presenting with a malignancy and raised hCG, with reliable detection of GTT when hCG is ≥1497 IU/L. This technology can ensure that suspected GTT cases are confirmed without the need for potentially dangerous biopsies and patients with non-GTT malignancies can be spared aggressive non-curative therapies.

## Methods

### Sample collection and extraction

The human samples, collected with informed written consent, used in this research project were obtained from the Imperial College Healthcare Tissue Bank (ICHTB), which is approved by Wales REC3 to release human material for research (17/WA/0161), and the samples for this project (R14021, R19029) were issued from sub-collection reference numbers ONC_MS_11_003, MED_RF_19_013 and CAN_GM_19_034. Cases with tumours of known origin refer to those 1) whose tumour arose during hCG monitoring following a molar pregnancy, 2) with genetic diagnosis from their tumour tissue, 3) with non-host alleles previously identified by STR genotyping of cfDNA (Table 1). Cases of unknown origin lacked all of the above, although in some cases they subsequently had tumour tissue removed and a genetic diagnosis was made.

Blood samples were stored in EDTA tubes on ice for a maximum of 2 h before plasma was isolated: after centrifugation at 1000 *g* for 10 min at 4 °C the supernatant was isolated and centrifuged at 2000 *g* for 10 min at 4 °C^25^. The supernatant was removed and then stored at −80 °C until cfDNA was extracted from 3 ml of plasma using the QIAamp circulating nucleic acid kit (Qiagen) in accordance with the manufacturer’s instructions. Genomic DNA was extracted from the buffy coat of the centrifuged sample or from a separate whole blood sample using the QIAamp DNA Blood Mini kit (Qiagen). Where available tumour tissue was manually dissected from formalin-fixed paraffin embedded (FFPE) tissue sections and DNA was extracted using a using a QIAamp DNA FFPE Tissue Kit (Qiagen, UK) according to the manufacturer’s instructions. DNA was quantified using Qubit fluorometry (Thermo Fisher).

### DNA library preparation

Typically, 20 ng (range 10–100 ng) of cfDNA or gDNA was used to prepare a DNA library in accordance with the manufacturer’s instructions, using Cell3™Target Library Preparation kit (Nonacus) or Lotus DNA Library Prep Kit (Integrated DNA Technologies), respectively. Illumina dual-indexed adaptors containing nine nucleotide unique molecular identifiers (UMIs) were ligated, before PCR amplification with the number of cycles adjusted to input DNA (see Supplementary Table 1 for library preparation details). Following purification with Target Pure NGS Clean-up Beads (Nonacus), quality control using Tapestation High Sensitivity D5000 ScreenTape (Agilent Technologies) and quantification using Qubit fluorometry, samples were pooled (100–150 ng of cfDNA libraries and 40–50 ng of gDNA libraries). Pooled DNA libraries totalling ~1500 ng were hybridised to a 60 kb custom capture library, which contained 195 common autosomal SNPs (median minor allele 0.49; range 0.26–0.71; minimum of 3 and median of 8.5 SNPs per chromosome) from the Cell3 Target Paternity Panel (Nonacus), 7 chrX targets and 6 chrY targets. The regions of interest were captured and amplified (14 cycles) using the Cell3 Target Capture Enrichment Reagents kit (Nonacus). Amplified libraries were purified (Target Pure NGS Clean-up Beads), evaluated (Tapestation High Sensitivity D5000 ScreenTape), quantified (Qubit) and, if necessary, pooled before sequencing on Illumina NextSeq500 mid output using the following read lengths: R1 (71), R2 (71), i7 (17); i5 (8).

### Data processing and SNP genotyping

Fastq files for R1, R2 (UMI), R3, I1 and I2 were generated using bcl2fastq. Reads were aligned to hg38, using bwa-mem v0.7.13^26^ fgbio’s toolset was used to annotate the RX tag of the BAM file with the UMI (http://fulcrumgenomics.github.io/fgbio/tools/latest/AnnotateBamWithUmis.html). The ‘pileups snps’ tool in amplimap^27^ was used to generate counts for all reference and alternate autosomal SNPs. To remove PCR duplicates and to correct PCR and sequencing errors, reads with a minimum mapping quality of Q20 which had identical UMIs and mapping location were grouped. Consensus calls were generated for each group: only bases with a minimum quality of Q20 comprising >80% of the total family of at least two reads were counted. Non-host alleles (alleles present in cfDNA that did not match the homozygous alleles in the patient) with a minimum count of two non-host bases per SNP were identified. The non-host cfDNA fraction was calculated as the mean fraction of consensus base counts for non-host SNP divided by the sum of the patient and non-host consensus base counts. For cases with detectable non-host DNA, read counts for sex chromosome regions were analysed to determine the sex of the tumour. B allele frequencies for SNPs that were heterozygous in the patients were used to identify features of the causative pregnancy in gestational cases and evidence of copy number alterations in non-gestational cases.

### STR genotyping

Fifteen autosomal STR loci on 13 chromosomes and the Amelogenin (sex chromosome) locus were amplified from 1–4 ng of DNA using the AmpFlSTR Identifiler Plus or Globalfiler IQC kits (Applied Biosystems, Warrington, UK) and resolved by capillary electrophoresis using an ABI 3130 Genetic Analyzer. Genotypes were analysed using GeneMapper version 5.0 software (Applied Biosystems, Warrington, UK).

### Correlation of serum hCG levels and non-host cfDNA

Spearman’s rank correlation coefficient was calculated for all gestational samples with ≥12 SNPs with non-host alleles using the cor.test() function in R.

### Reporting summary

Further information on research design is available in the Nature Research Reporting Summary linked to this article.

## Supplementary information

Supplemental Figures

Supplemental Table 1

Reporting Summary

## Data Availability

Sequencing data (.bam files) are available from the European Nucleotide Archive (accession number PRJEB49976).

## Code availability

Custom codes used in this study can be obtained upon a reasonable request to the corresponding author.
