# Supplementary material for: Sensitive screening of single nucleotide polymorphisms in cell free DNA for diagnosis of gestational tumours
Source: NPJ Genom Med. 2022 Apr 8;7:26. doi: 10.1038/s41525-022-00297-x (PMC8993869; doi:10.1038/s41525-022-00297-x)
Supplement: Supplementary file 1 — Supplemental Figures [file 41525_2022_297_MOESM1_ESM.docx]

Supplemental Figures for

**Sensitive screening of single nucleotide polymorphisms in cell free DNA for diagnosis of gestational tumours**

Geoffrey J Maher ^1,2^, Rosemary A Fisher ^1^, Baljeet Kaur ^1^, Xianne Aguiar ^1^, Preetha Aravind ^1^, Natashia Cedeno ^1^, James Clark ^1^, Debbie Damon ^1^, Ehsan Ghorani ^1^, Adam Januszewski ^1^, Foteini Kalofonou ^2^, Ravindhi Murphy ^1^, Rajat Roy ^1,2^, Naveed Sarwar ^1^, Mark R Openshaw ^1^, Michael J Seckl^1,2^

^1^ Trophoblastic Tumour Screening & Treatment Centre, Imperial College London, Charing Cross Campus, Fulham Palace Road, London, W6 8RF, UK

^2^ Department of Surgery and Cancer, ICTEM Building, Hammersmith Hospitals Campus of Imperial College London, Du Cane Road, London W12 0NN, UK

| 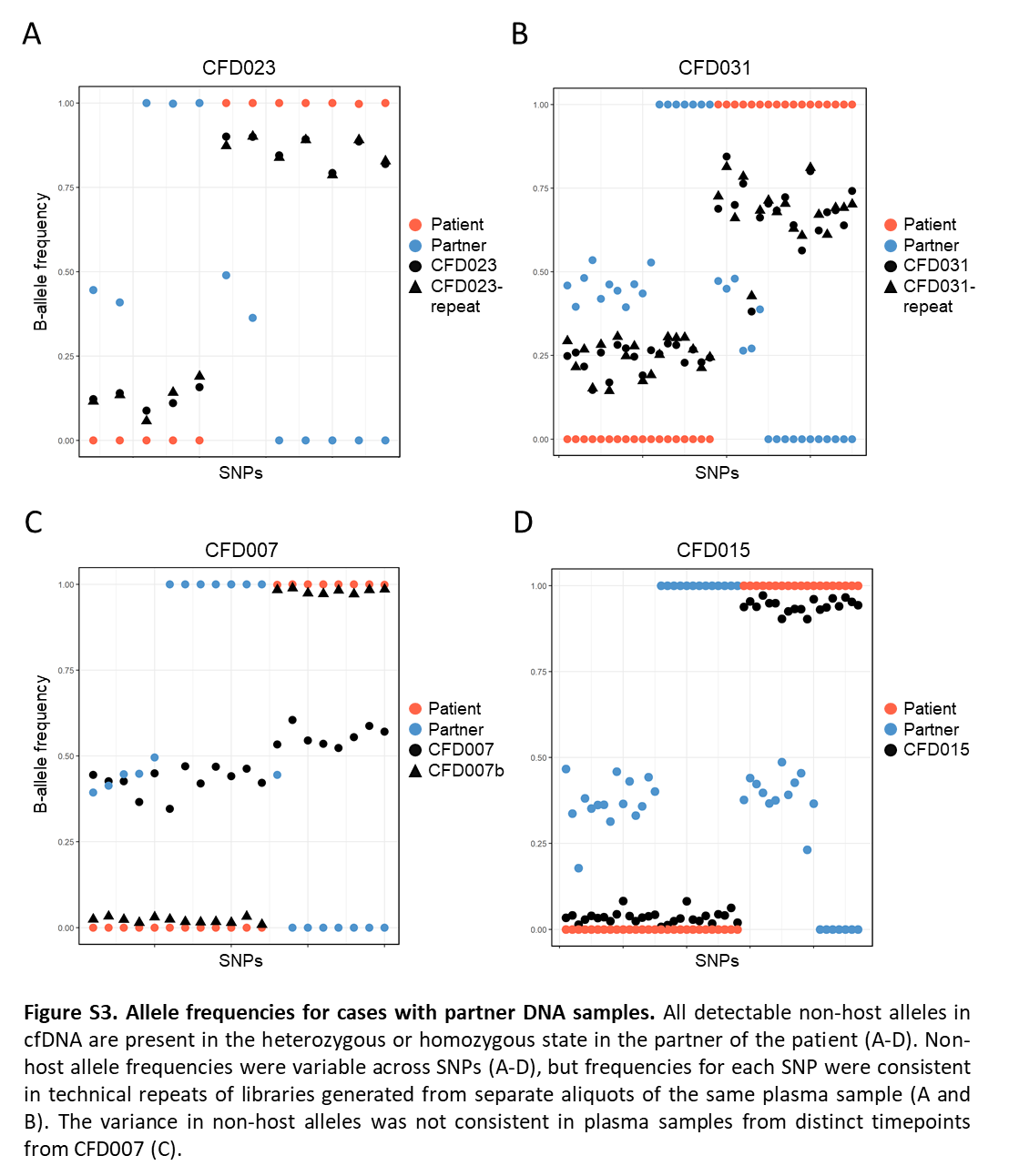 |
| --- |
| **Supplementary Figure 1. Allele frequencies for cases with partner DNA samples.** All detectable non-host alleles in cfDNA (black) are present in the heterozygous or homozygous state in the partner (blue) of the patient (red) (A-D). Non-host allele frequencies were variable across SNPs (A-D), but frequencies for each SNP were consistent in technical repeats of libraries generated from separate aliquots of the same plasma sample extracted 4 years apart (A and B). The variance in non-host alleles was not consistent in plasma samples from distinct timepoints from CFD007 (C). |

| 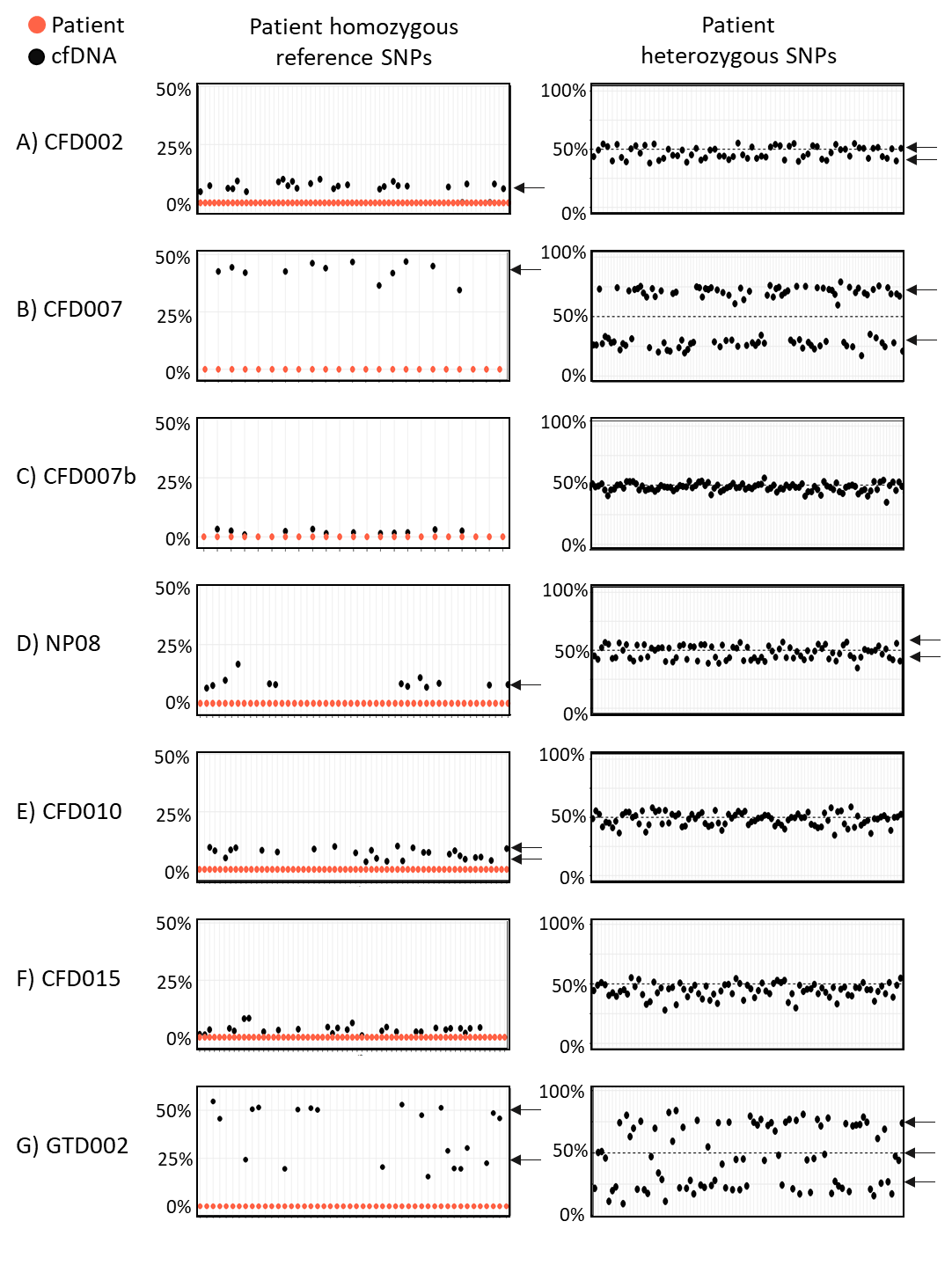 |
| --- |
| **Supplementary Figure 2. Examples of cfDNA B-allele frequency profiles from monospermic and dispermic molar pregnancies.** cfDNA from monospermic moles (A-D) have a unimodal distribution for non-host alleles at homozygous SNPs and a bimodal distribution at heterozygous SNPs, which may not be distinguishable at low fractions (e.g. CFD007b). cfDNA from dispermic molar pregnancies (E-G) have a bimodal distribution for non-host alleles at homozygous SNPs and more than two modes at heterozygous SNPs, which may not be distinguishable at low fractions (e.g. CFD015) |

| 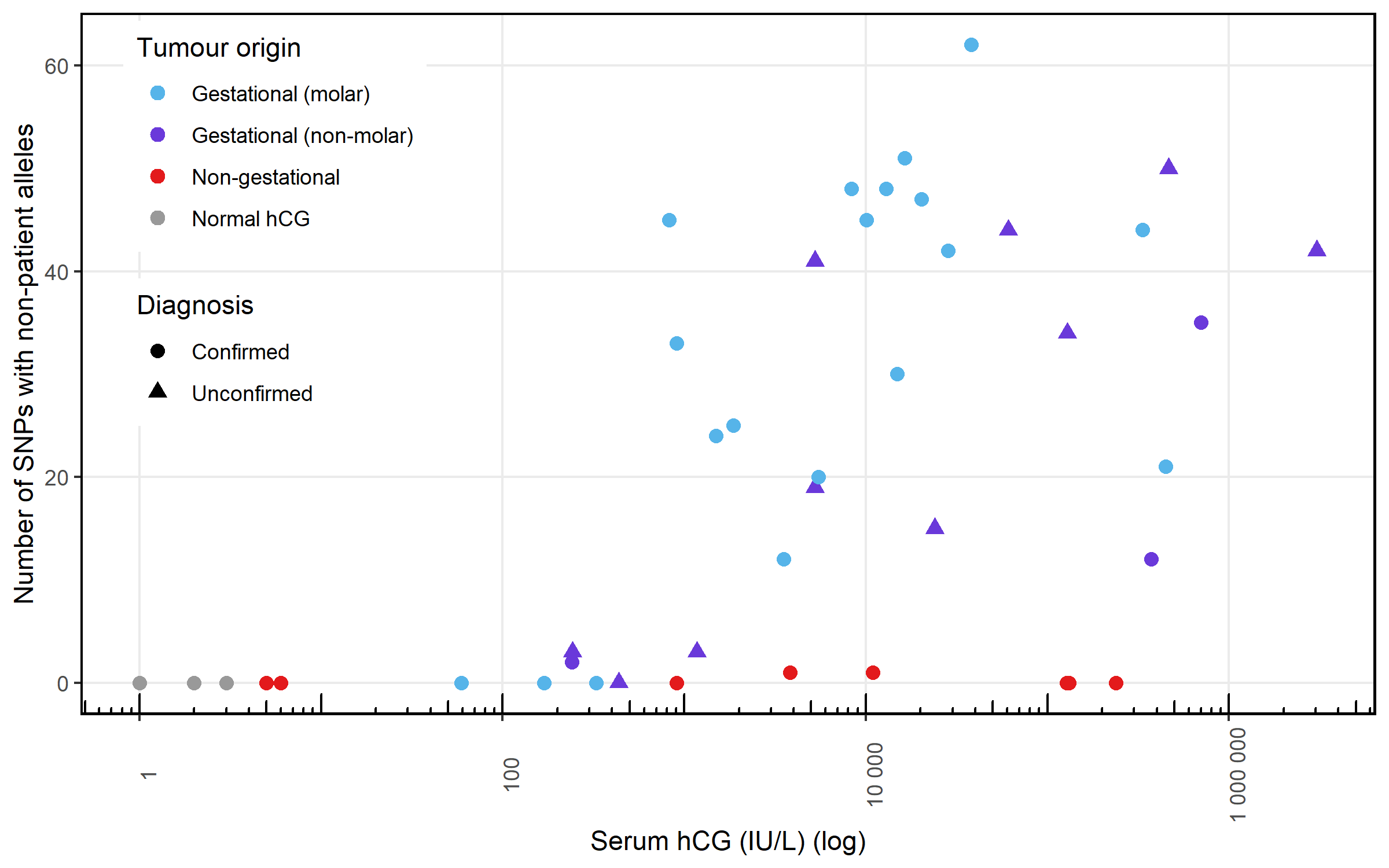 |
| --- |
| **Supplementary Figure 3. Number of SNPs with non-host alleles in all cfDNA samples.** Samples from patients with non-gestational tumours or normal hCG levels had 0 or 1 SNPs with non-host alleles. In gestational cases, ≥12 SNPs were detected in all cases when the hCG was ≥1497 IU/L, but detection below this hCG level was variable. Confirmed = cases of known origin. Unconfirmed = cases that did not have tissue available to confirm the diagnosis. |

| 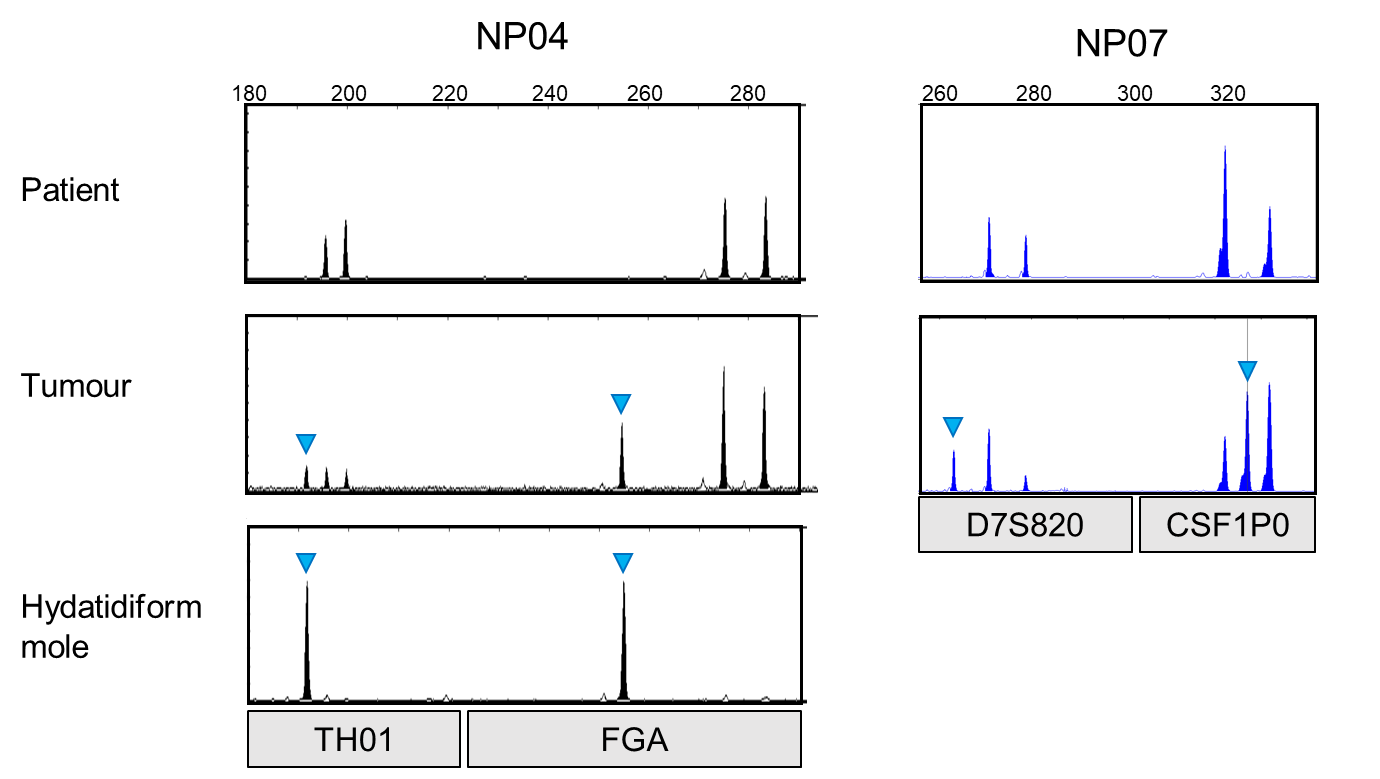 |
| --- |
| **Supplementary Figure 4. Short tandem repeat genotyping of tumours from NP04 and NP07.** Two representative loci are demonstrated for each patient, with the x-axis representing the size of the DNA fragments generated and the y-axis representing arbitrary units of fluorescent intensity. The presence of non-patient alleles (blue arrowheads) in the tumour samples confirms the gestational origin. For NP04, identical alleles were found in the patient’s previous complete hydatidiform mole. |

| 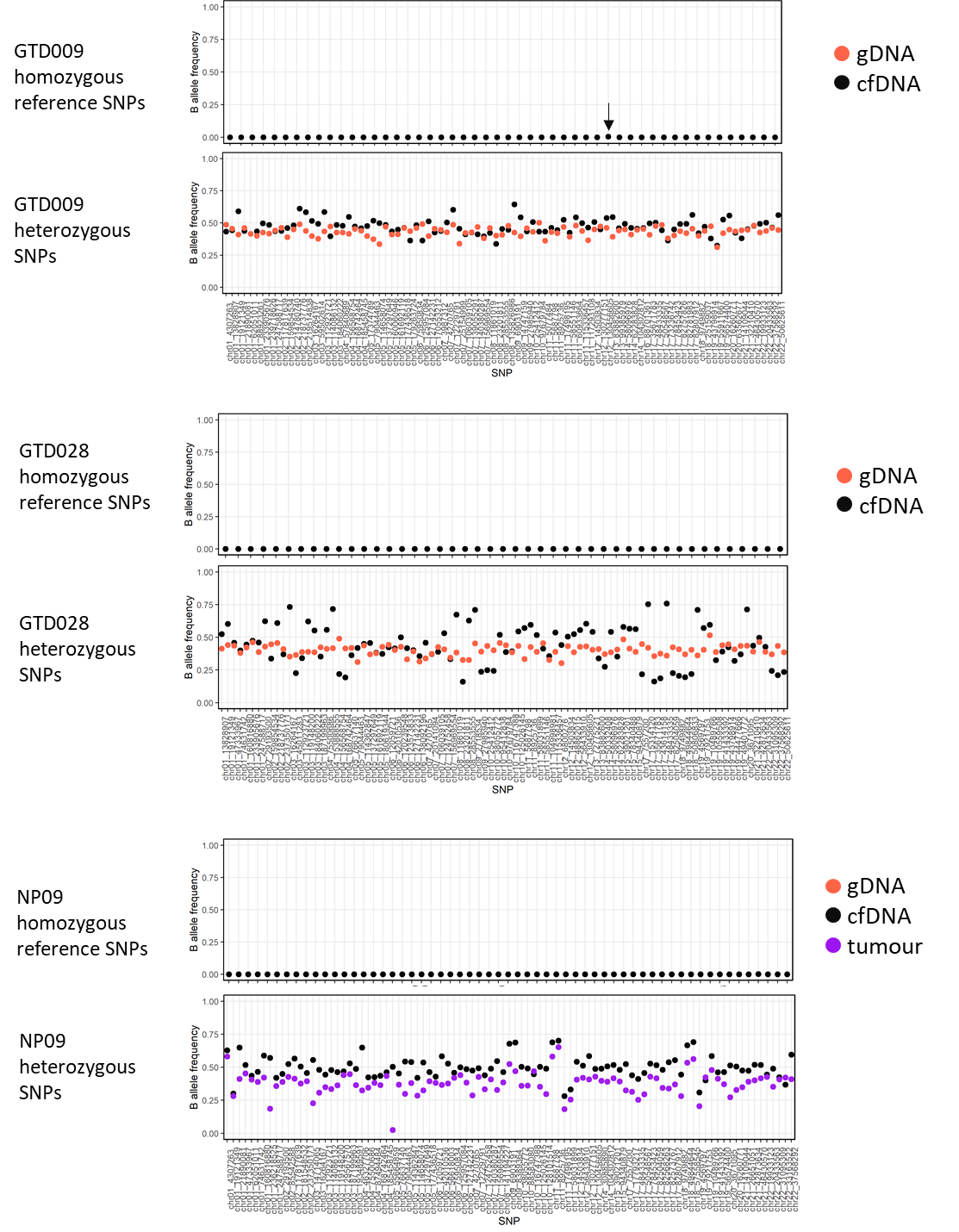 |
| --- |
| **Supplementary Figure 5. Evidence for copy number alterations in non-gestational tumour cfDNA.** Upper panels for each patient show the absence of non-host alleles at SNPs for which the patient is homozygous. A single SNP in RC009 has a non-host allele at 0.5% (arrow). Lower panels for each patient show the B allele frequency for heterozygous SNPs, revealing deviations from the patients’ gDNA sample. For NP09, the B allele profile of the cfDNA was largely consistent with that of the matched tumour sample. |

| 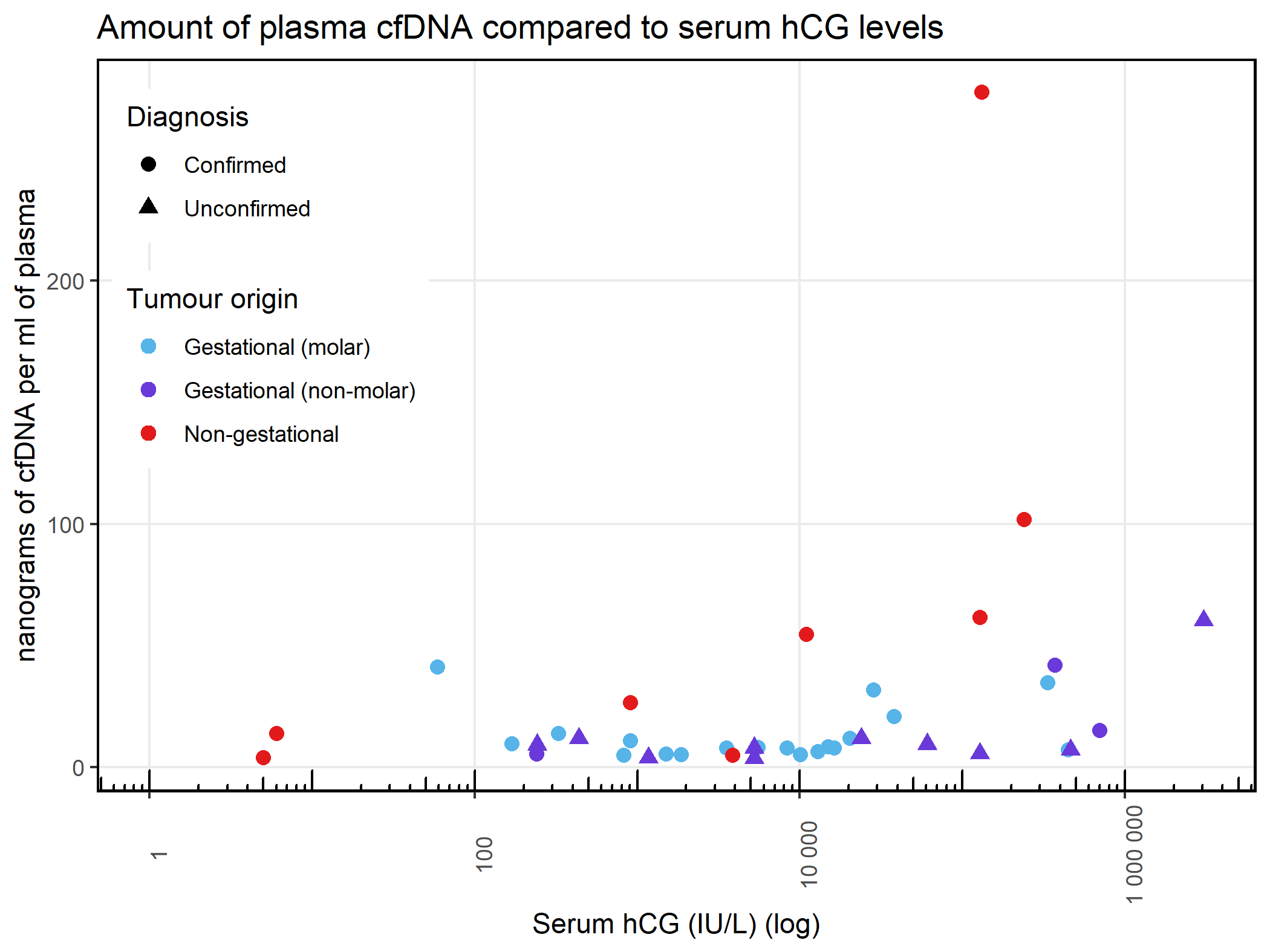 |
| --- |
| **Supplementary Figure 6. Amount of cfDNA per ml of plasma in patients with elevated serum hCG.** Confirmed = cases of known origin. Unconfirmed = cases with non-host cfDNA that did not have tissue available to confirm the diagnosis. |
