# Supplementary material for: Sensitive screening of single nucleotide polymorphisms in cell free DNA for diagnosis of gestational tumours
Source: NPJ Genom Med. 2022 Apr 8;7:26. doi: 10.1038/s41525-022-00297-x (PMC8993869; doi:10.1038/s41525-022-00297-x)
Supplement: Supplementary file 3 — Reporting Summary [file 41525_2022_297_MOESM3_ESM.pdf]

nature portfolio

Corresponding author(s): Geoffrey J Maher

Last updated by author(s): Oct 27, 2021

Reporting Summary

Nature Portfolio wishes to improve the reproducibility of the work that we publish. This form provides structure for consistency and transparency in reporting. For further information on Nature Portfolio policies, see our Editorial Policies and the Editorial Policy Checklist.

Statistics

For all statistical analyses, confirm that the following items are present in the figure legend, table legend, main text, or Methods section.

n/a	Confirmed	
☒	☐	The exact sample size (*n*) for each experimental group/condition, given as a discrete number and unit of measurement
☒	☐	A statement on whether measurements were taken from distinct samples or whether the same sample was measured repeatedly
☒	☐	The statistical test(s) used AND whether they are one- or two-sided<br>*Only common tests should be described solely by name; describe more complex techniques in the Methods section.*
☒	☐	A description of all covariates tested
☒	☐	A description of any assumptions or corrections, such as tests of normality and adjustment for multiple comparisons
☒	☐	A full description of the statistical parameters including central tendency (e.g. means) or other basic estimates (e.g. regression coefficient) AND variation (e.g. standard deviation) or associated estimates of uncertainty (e.g. confidence intervals)
☒	☐	For null hypothesis testing, the test statistic (e.g. *F*, *t*, *r*) with confidence intervals, effect sizes, degrees of freedom and *P* value noted<br>*Give P values as exact values whenever suitable.*
☒	☐	For Bayesian analysis, information on the choice of priors and Markov chain Monte Carlo settings
☒	☐	For hierarchical and complex designs, identification of the appropriate level for tests and full reporting of outcomes
☒	☐	Estimates of effect sizes (e.g. Cohen's *d*, Pearson's *r*), indicating how they were calculated

Our web collection on statistics for biologists contains articles on many of the points above.

Software and code

Policy information about availability of computer code

Data collection	No software was used
Data analysis	amplimap (doi: https://doi.org/10.1093/bioinformatics/btz905; https://github.com/koelling/amplimap) was used to count the number of reads for reference alternate alleles.<br>Custom R code was used to analyse the output from amplimap and determine the genotype of each SNP in cell free DNA and genomic DNA

For manuscripts utilizing custom algorithms or software that are central to the research but not yet described in published literature, software must be made available to editors and reviewers. We strongly encourage code deposition in a community repository (e.g. GitHub). See the Nature Portfolio guidelines for submitting code & software for further information.

Data

Policy information about availability of data

All manuscripts must include a data availability statement. This statement should provide the following information, where applicable:

- Accession codes, unique identifiers, or web links for publicly available datasets
- A description of any restrictions on data availability
- For clinical datasets or third party data, please ensure that the statement adheres to our policy

The sequencing data will be submitted to the European Nucleotide Archive (ENA) before publication.

Field-specific reporting

Please select the one below that is the best fit for your research. If you are not sure, read the appropriate sections before making your selection.

☒ Life sciences ☐ Behavioural & social sciences ☐ Ecological, evolutionary & environmental sciences

For a reference copy of the document with all sections, see nature.com/documents/nr-reporting-summary-flat.pdf

Life sciences study design

All studies must disclose on these points even when the disclosure is negative.

Sample size	Cases of hCG secreting tumours, in particular non-gestational hCG-secreting tumours, are rare and no sample-size calculation was performed
Data exclusions	No data exclusions
Replication	After analysing a validation set of samples of known origin, a further set of test samples was analysed
Randomization	No randomization was performed. Samples were analysed with relation to the origin of their tumours and their hCG levels
Blinding	Analysis of the validation set was not blinded. In the test set, analysis was performed before the diagnosis was known

Reporting for specific materials, systems and methods

We require information from authors about some types of materials, experimental systems and methods used in many studies. Here, indicate whether each material, system or method listed is relevant to your study. If you are not sure if a list item applies to your research, read the appropriate section before selecting a response.

Materials & experimental systems

n/a	Involved in the study	
☒	☐	Antibodies
☒	☐	Eukaryotic cell lines
☒	☐	Palaeontology and archaeology
☒	☐	Animals and other organisms
☐	☒	Human research participants
☒	☐	Clinical data
☒	☐	Dual use research of concern

Methods

n/a	Involved in the study	
☒	☐	ChIP-seq
☒	☐	Flow cytometry
☒	☐	MRI-based neuroimaging

Human research participants

Policy information about studies involving human research participants

Population characteristics	Female patients with hCG-secreting tumour and/or germ cell tumours
Recruitment	Patients were recruited to donate samples to the Imperial College Healthcare Tissue Bank (ICHTB). Although patients with a variety of hCG levels were initially recruited, patients specifically with low or normal hCG levels were recruited to act as appropriate controls
Ethics oversight	Human samples used in this research project were obtained from the Imperial College Healthcare Tissue Bank (ICHTB), which is approved by Wales REC3 to release human material for research (17/WA/0161)

Note that full information on the approval of the study protocol must also be provided in the manuscript.
